# Malignant Cerebral Edema following CT Myelogram Using Isovue-M 300 Intrathecal Nonionic Water-Soluble Contrast: A Case Report

**DOI:** 10.1155/2011/212516

**Published:** 2011-01-13

**Authors:** Brian C. Kelley, Simon Roh, Philip L. Johnson, Paul M. Arnold

**Affiliations:** ^1^Department of Neurosurgery, University of Kansas Medical Center, 3901 Rainbow Boulevard MS 3021, Kansas City, KS 66160, USA; ^2^School of Medicine, University of Kansas Medical Center, Kansas City, KS 66160, USA; ^3^Department of Radiology, University of Kansas Medical Center, Kansas City, KS 66160, USA

## Abstract

Lumbar myelogram utilizing nonionic contrast is a commonly performed procedure to identify spinal pathology. Complication rates are low. Cerebral edema has been shown to occur following intrathecal injection of ionic contrast; however, no current literature has documented this complication relating to the ubiquitously used nonionic contrast medium. We report a case of a patient who developed malignant cerebral edema following a lumbar myelogram with Isovue-M 300 nonionic water-soluble intrathecal contrast. We believe this is the first reported case of cerebral edema resulting from the use of a nonionic contrast.

## 1. Introduction

The neurotoxicity of inadvertent intrathecal injection of ionic contrast media during myelography is well documented. To our knowledge, there are no known case reports detailing malignant cerebral edema as a response to lumbar myelography using nonionic contrast media. We report a patient who displayed altered mental status and radiographic evidence of severe brain edema following myelography using nonionic contrast. 

## 2. Case Report

A 50-year-old African-American woman presented with increased somnolence, headache, vision changes, and nausea. One day prior she had undergone a L2-L3 interspace lumbar puncture in the prone position using a 25-gauge spinal needle with infusion of 10 mL of Isovue-300 nonionic contrast at a concentration of 61.2 grams iopamidol/100 mL equivalent to 300 mg iodine/mL. The patient was placed prone following the myelogram. There was no history of new medication, trauma, or complications during her recent procedure. 

At presentation, the patient denied meningismus, photophobia, or seizure activity. Past medical history was positive for cervical spondylosis, hyperlipidemia, hypertension, and mitral valve regurge. She was considered to be in good health prior to the procedure. Surgical history included a C3–C6 anterior cervical fusion four months prior to presentation. She was complaining of neck and upper extremity pain, which was the indication for the myelogram. Her only reported allergy was to Naproxen. Current medication included aspirin, Colace, Cymbalta, Neurontin, Vicodin, Lisinopril, Prilosec, Senokot, Zocor, and Ultram. 

**Figure 1:**
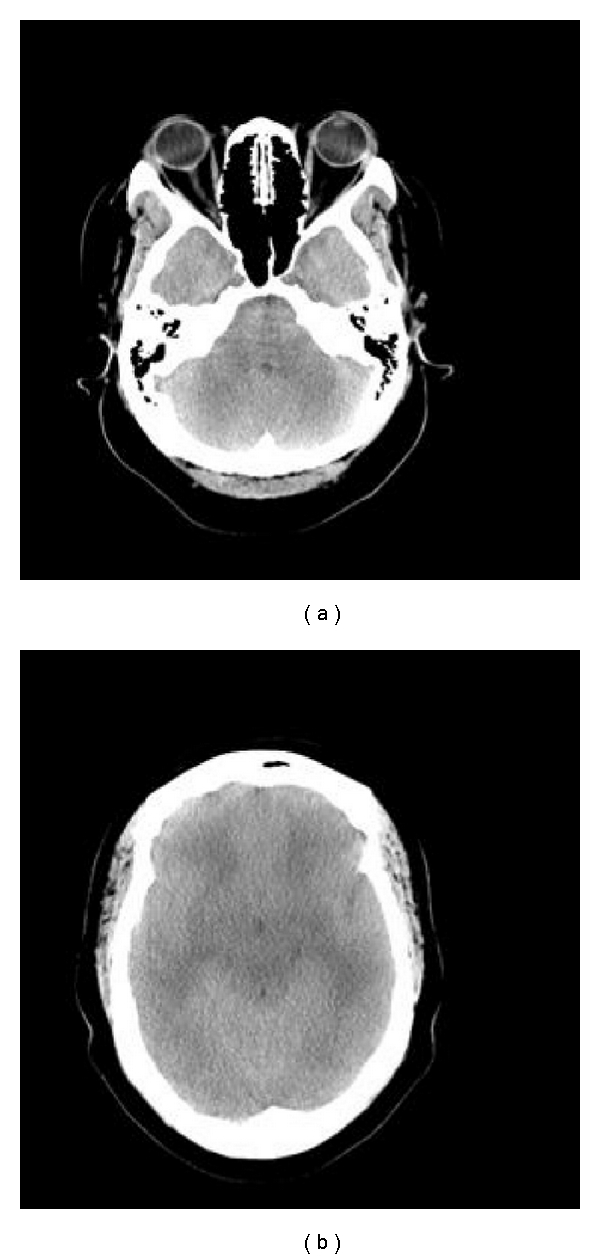
(a, b) Axial CT scans demonstrating diffuse cerebral edema on admission.

Following the myelogram, the patient was observed for six hours in the postprocedure area. She was discharged in stable condition, with no complaints. She returned to the emergency room six hours later, complaining of a postural headache, which progressed to diminished mental status. A physical exam was positive for lethargy, absent spontaneous retinal venous pulsation, and sluggishly reacting pupils bilaterally. Her vitals as well as standard laboratory values were all within normal limits. Computed tomography (CT) scan of the head demonstrated marked bihemispheric cerebral edema with obliteration of the basal cisterns and cerebral sulci (Figure 1).

The patient was admitted to the neurosurgical intensive care unit and was immediately started on dexamethasone and mannitol. Serial CT scans gradually showed edema resolution corresponding to the patient's slow return to baseline neurological exam. She was discharged on hospital day five with a decadron taper. Her neurologic examination and sensorium was normal two weeks after the procedure and remains so one year after the event. 

## 3. Discussion

Cerebral edema is defined as an increase in brain water content above the normal content of approximately 80% and is invariably a response to a primary brain insult [1]. Cerebral edema can lead to increased intracranial pressure as well as movement of the intracranial contents, which can cause injury by compression and mechanical manipulation. When cellular function cannot be sustained due to inadequate delivery of nutrients, such as that caused by global ischemia resulting from massive edema, a series of biochemical reactions called the ischemic cascade begins within the cerebrum. 

Signs and symptoms of cerebral edema can vary widely. Some of the more common findings in patients include abnormal response to pain, decorticate or decerebrate posture, altered mentation, sustained heart rate deceleration (more than 20 beats/minute (bpm)), vomiting, headache, and lethargy or not being easily awakened [2]. Close bedside monitoring is required, focusing on the level of consciousness and new or worsening focal neurological deficits; the patient may need to be admitted to the intensive care unit [1]. Serial CTs and MRIs may confirm worsening of cerebral edema, intracranial compartmental and midline shifts, herniation syndromes, and ischemic brain injury. This neuroimaging can also help differentiate the type of edema present, whether focal or global, and involvement of gray or white matter [1, 3]. 

Lumbar myelography is a common procedure used to evaluate spinal pathology. Its use has declined over the past decade due to the availability of high-fidelity magnetic resonance imaging (MRI) [4]. Myelography is considered safe, having a low risk of seizures, contrast reactions, bleeding, or other postmyelography complications [4]. Adverse reactions to contrast agents can be mild to life threatening; the contrast agents most likely to cause adverse reactions are those with higher osmolality, and ionic agents have two times more osmolality than nonionic agents [5]. However, low-osmolality agents cost up to 10 times more than high-osmolality ionic agents [5]. The ionic nature of a contrast medium is the primary cause of neurotoxicity; other elements involved include the dosage and concentration, the duration of exposure, and its osmolality [6]. There are numerous well-documented cases reporting seizures and encephalopathy with use of metrizamide [7], which was introduced in 1969 as the first nonionic water soluble contrast. Later development of iohexol and iopamidol in the late 1970s replaced metrizamide due to a lower risk of adverse neurological effects [8–10]. In 1986, Elkin et al. reported no cases of mental status changes or seizures in 248 patients after iohexol myelography [9]. In 1988, two large case series of iohexol lumbar myelographies were reported, and no serious neurologic complications were observed; in addition, the side effects of headache, nausea, and vomiting were postulated to be the result of the spinal puncture itself rather than the contrast medium [11, 12]. Torvik and Walday noted that specific contrast-induced mechanisms may indeed be partly responsible for the headache and vomiting, because those symptoms occur with different frequencies after injection of various types of contrast media [10]. A small number of case reports discussing the use of nonionic mediums document seizures [13–21], aseptic meningoencephalitis [22–25], aseptic meningitis [26–29], and encephalopathy [7, 30].

Several case reports have documented neurotoxicity following angiography using nonionic contrast media, including encephalopathy [31, 32], cerebral edema [32], selective cortical injury [33], and transient partial amnesia [34]. Donaghy et al. suggested that disruption of the blood-brain barrier occurs during angiographic use of nonionic contrast, allowing diffusion into the parenchyma, thus exerting a neuronal toxic effect resulting in acute encephalopathy [30]. Frontera et al. reported a case of contrast-induced neurotoxicity and postulate that the toxicity may have been exacerbated by a slower washout of the CM, and there may have been an immune-mediated hypersensitivity mechanism as well as selective cortical sensitivity to contrast materials. In addition, the authors theorize that delayed apoptotic infarction may have played a role in the development of the neurotoxicity [33]. Sawaya et al. reported a case of contrast-induced encephalopathy, theorizing it secondary to seepage through the BBB by mechanisms not including the contrast osmolality; this seepage then resulted in temporary disruption of the central cholinergic synaptic transmission [31]. Guimaraens et al. reported a case of cerebral edema and transient encephalopathy caused by the use of nonionic CM in angiography for treatment of a cerebral aneurysm. The authors consider the neurotoxicity as secondary to the volume of contrast medium used, as well as to the patient's history of hypertension, which predisposed him to disruption of the BBB and cerebral autoregulatory dysfunction [32]. 

The neurotoxicity of inadvertent intrathecal injection of ionic contrast media during myelography is well documented [35–54]. There is a paucity of the literature, however, concerning the pathophysiology of the rare but severe adverse neurological conditions that have been attributed to the use of intrathecal injection of nonionic contrast media.

As various neurotoxic effects can be seen in both rodents and humans, neurotoxic studies in rodents may have predictive value for neurotoxic effects in humans [55]. In their study of adult rabbits having had intrathecal injection of both ionic and nonionic contrast media, Lee et al. found subpial edema as well as acute and chronic inflammation; these conditions were most severe after ionic injection and milder after nonionic injection [56]. Miyazawa et al. infused rat blood with an ionic CM, which deformed red blood cells and in turn induced cerebral edema, destruction of the BBB, and animal death. The infusion of nonionic CM into rat blood had less effect on the red blood cells, and no cerebral damage was seen in these rats [57]. Mennini et al. compared the binding properties of two nonionic CM in vitro on select rat brain areas to evaluate whether CM-related neurotoxicity derived from their interactions with specific receptors on neural membranes. The authors concluded that the nonionic CM did not influence normal neural membrane functions; nonionic CM had no effect on the binding capacities of the receptors for common neurotransmitters. The authors postulate that the occasional neurotoxic effects seen with nonionic CM do not occur as a consequence of specific action on brain receptors, but that nonionic CM may have an indirect, postmembrane site of action. The authors determined it unlikely that nonionic CM exert their neurotoxic effects through specific binding to neurotransmitter receptors [58]. Wible Jr. et al. compared the neurotoxicity of four nonionic contrast materials, similar in osmolality and viscosity, injected into 124 adult rats. The authors found that the nonionic material with smaller degrees of hydrophilicity tested (iopromide) produced the highest degree of neurotoxicity [59]. Luzzani et al. compared the acute toxicities of monomer and dimer nonionic contrast media injected into mice and rats to evaluate the determinants of neurotoxicity. The median lethal dose values obtained by Luzzani et al. were similar to those obtained by Wible et al. and Luzzani et al. concluded that the neurotoxicity of nonionic CM is due more to characteristics of chemical structure other than hydrophilicity, than on the physicochemical characteristics of their solutions. In other words, the neurotolerability of nonionic CM depends more on the chemical structure of the CM than on the physicochemical characteristics of the CM. Differences in hydrophilicity between CM molecules did not account for the differences in their neurotolerability [55]. 

Current theories of the pathophysiology of neurotoxicity due to intrathecal use of nonionic contrast include direct neurotoxicity [23], serum osmolarity differences, [30] and lipid solubility of the agent [60]. According to guidelines by the American College of Radiology, patients with any of the following listed conditions should be considered for low-osmolality nonionic contrast agents: advanced age, food or medication allergies, hay fever or asthma, previous anaphylactoid reaction to contrast material, treatment with nephrotoxic agents, preexisting renal insufficiency, or multiple medical problems or an underlying disease [61]. Our patient had none of these conditions, nor did she have any identifiable risk factors for seizure, encephalitis, or hemorrhage. We postulate that the mechanism for our patient's acute onset of massive cerebral edema is due to the dysfunction of the patient's BBB, with subsequent loss of autoregulation of cerebrovascular blood flow and resulting vasogenic edema from hyperemia. Further elucidation of the mechanisms responsible for the development of severe neurological adverse events is needed.

### 3.1. Treatment

 Treatment of vasogenic edema includes both steroid administration and osmotherapy [1]. The osmotic gradient created by osmotherapy induces the movement of water from the brain extracellular compartment into the vasculature; this then improves intracranial elastance and decreases intracranial volume [1]. In a normal individual, brain volume makes up 80%, blood volume makes up 10%, and CSF volume makes up 10% [1, 62]. The traditional recommendation for patients with increased intracranial pressure is osmolality in the range of 300–320 mOsm/L [1, 62, 63]. 

An osmotic agent is considered ideal if it is inert, nontoxic, has few systemic side effects, and creates a favorable osmotic gradient by remaining in the intravascular compartment [1, 62, 64–66]. Another important characteristic of an ideal osmotic agent is the ability of an intact BBB to exclude the agent, quantified by the reflection coefficient *σ*; the range of the potential coefficients is 0 (freely permeable) to 1 (entirely excluded). The risk of rebound cerebral edema is greater as the BBB permeability of the agent increases [67]. Osmotic agents with *σ* approaching 1 are completely excluded by an intact BBB and are less then likely to cause rebound edema [1, 65, 66]. 

Rebound cerebral edema may occur with mannitol (*σ* = 0.9) use, because in areas in which the BBB is not intact, the osmotic gradient is reversed between the brain and the intravascular compartment [1, 64]. Studies have shown that mannitol appears in the CSF at approximately 12% of the corresponding plasma concentration eight hours after IV administration [1, 68], and rebound increases in ICP with mannitol use are well documented [1]. Glycerol (*σ* = 0.48) and urea (*σ* = 0.59) have transient osmotic effects and are only partly excluded by the intact BBB; therefore, they are not ideal agents for osmotherapy [1]. Sodium chloride has been proposed as a more effective osmotic agent because it has a reflection coefficient of 1.0 [1, 64, 69]. 

No guidelines exist for ICP monitoring in cerebral edema attributed to intrathecal drug administration; rather, decisions regarding ICP monitoring are often based on results of neuroimaging studies as well as the neurological status of the patient [1]. An algorithmic approach is utilized in the medical management of cerebral edema with or without elevated ICP. General measures include avoiding dehydration and systemic hypotension, maintaining normal body temperature, and positioning the head and neck so that intracranial venous outflow is facilitated. Specific therapeutic interventions include osmotherapy, controlled hyperventilation, administering corticosteroids or diuretics, and suppressing cerebral metabolism with pharmacology [1].

## 4. Conclusion

We present a case of malignant cerebral edema following CT myelogram using a nonionic water-soluble iodinated contrast agent administered intrathecally. This is an unheard-of complication with the use of a nonionic contrast agent. Clinicians should be aware of this rare, but potentially fatal, complication of this commonly performed procedure.
